# Mobile Health App for Prostate Cancer Patients on Androgen Deprivation Therapy: Qualitative Usability Study

**DOI:** 10.2196/20224

**Published:** 2020-11-03

**Authors:** Junaid Nabi, Eugene B Cone, Anjali Vasavada, Maxine Sun, Kerry L Kilbridge, Adam S Kibel, Donna L Berry, Quoc-Dien Trinh

**Affiliations:** 1 Division of Urological Surgery Department of Surgery Brigham and Women’s Hospital, Harvard Medical School Boston, MA United States; 2 Center for Surgery and Public Health Department of Surgery Brigham and Women’s Hospital, Harvard Medical School Boston, MA United States; 3 Lank Center for Genitourinary Oncology Dana-Farber Cancer Institute Boston, MA United States; 4 Phyllis F. Cantor Center Dana-Farber Cancer Institute Boston, MA United States

**Keywords:** mobile health application, prostate cancer, androgen deprivation therapy, thematic analysis, qualitative methods

## Abstract

**Background:**

Androgen deprivation therapy (ADT) increases the risk of metabolic adverse effects among patients with prostate cancer. The transformative impact of mobile health (mHealth) apps may benefit men managing activity and nutrition at home.

**Objective:**

This study aimed to evaluate the usability and patient experience of a newly developed mHealth app among prostate cancer patients on ADT and physicians’ beliefs about the potential benefits of using this app.

**Methods:**

This study took place over 2 months, beginning in March 2019. A sample of 5 patients (age 45-75 years) initiating ADT participated in a semistructured focus group discussion with a facilitator. The study participants also included 5 specialist physicians who provided in-depth interviews. An institutional review board–approved script was used to guide both the focus group and physician interviews. Usability was tested through specific scenarios presented to the patients, including downloading the mHealth app, entering information on physical activity and meals, and navigating the app. The focus group and interviews were audio recorded and transcribed. Content analysis was used to analyze the transcripts iteratively and exhaustively. Thematic discrepancies between reviewers were resolved through consensus.

**Results:**

The mean age of the patients was 62 years. This group included 4 White and 1 Latin American patients. The physician specialists included 2 urologists, 2 medical oncologists, and 1 radiation oncologist. Analyses revealed that the patients appreciated the holistic care enabled by the app. Difficulties were observed with registration of the app among 60% (3/5) of the patients; however, all the patients were able to input information about their physical activity and navigate the options within the app. Most patients (4/5, 80%) were able to input data on their recent meal. Among the health care physicians, the dominant themes reflected in the interviews included undermining of patients ability to use technology, patients’ fear of technology, and concern for the ability of older patients to access technology.

**Conclusions:**

The patients reported an overall positive experience of using an mHealth app to record and track diet and exercise. Usability was observed to be an important factor for adoption and was determined by ease of registration and use, intuitive appearance of the app, and focus on holistic cancer care. The physicians believed that the app was easy to use but raised concerns about usability among older men who may not typically use smartphone apps.

## Introduction

Health care is more efficient and effective when patients are actively engaged in their treatment [1]. Physicians and institutions perceive mobile health (mHealth) technologies—defined as the use of portable devices such as smartphones or tablets for health purposes—as an ideal tool to engage their patients [2]. This technology has already had a transformative impact on the delivery of care [3]. For example, home monitoring has been shown to be effective in reducing cancer symptom distress and improving blood pressure control [4,5]. Additionally, measurements obtained at home can be transmitted wirelessly to electronic medical records or directly to the clinician, allowing for rapid feedback and timely office visits triggered by abnormal values [6].

mHealth technologies are currently being used for a variety of medical conditions, including diabetes, asthma, and chronic obstructive pulmonary disease. With features such as frequent monitoring of patients, active collection of data, and seamless transmission of clinical information into electronic medical records, mHealth apps can enable real-time feedback and lead to improved communication with health care providers [6]. These aspects present a unique opportunity to use mHealth technology as a tool to prevent or alleviate adverse effects of disease and treatment, especially among prostate cancer patients.

While androgen deprivation therapy (ADT) is a standard treatment regimen for intermediate- or high-risk localized disease and metastatic or recurrent prostate cancer, the ensuing hypogonadal state results in significant metabolic and cardiovascular adverse events. Previous studies have documented that men exposed to gonadotropin-releasing hormone agonists—a common form of ADT—manifested a weight gain of 2.4%, an increase in body fat percentage of 9.4%, and a loss in lean body mass of 2%-4% (also known as sarcopenia) by the end of the first year [7,8]. We developed an mHealth app that provides dietary and exercise interventions to monitor and mitigate these metabolic disturbances. The objective of this study was to heuristically evaluate the usability and patient experience of this newly developed mHealth app among prostate cancer patients on ADT and physicians’ beliefs about the potential benefits of using this app.

## Methods

### Study Design

This usability study employed qualitative data collection and thematic content analysis methods. Focus group discussions and in-depth interviews were used for collecting data. An institutional review board (IRB)–approved script was used to guide these discussions. Thematic content analysis involves systematically coding and classifying textual information to explicate trends, patterns, frequency, of words, and their embedded relationships and discursive structures [9,10]. This technique enables researchers to understand the attributes of the content by lowering the level of interpretative complexity [11]. The Dana-Farber/Brigham and Women’s Cancer Center (DF/BWHCC) IRB approved this study.

### The mHealth App

**Figure 1:**
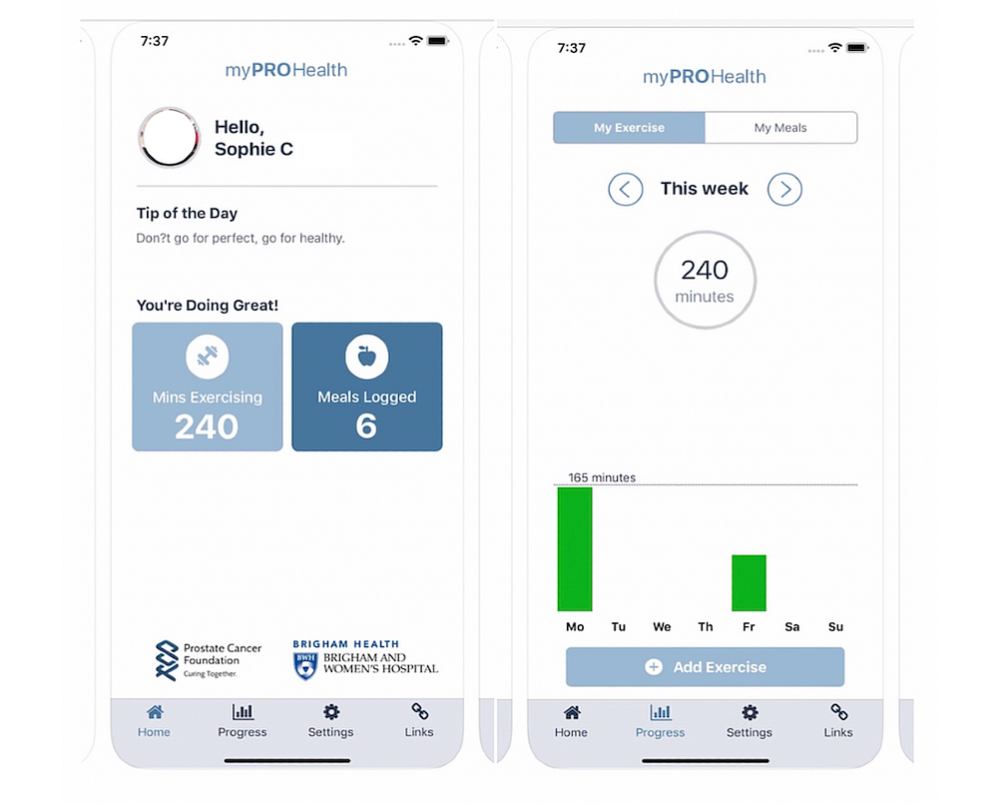
Screenshots of the mobile health application.

myPROHealth is a health and fitness tracking app developed by the Division of Urological Surgery at Brigham and Women’s Hospital. The app seeks to track, record, and promote exercise and good eating habits in men diagnosed with prostate cancer, who are about to initiate ADT. Figure 1 shows sample mHealth app interfaces. The features of the app enable patients to enter information on their recent physical activity and meals as well as monitor their progress.

### Study Setting and Focus Group Participants

Men diagnosed with prostate cancer, presenting to DF/BWHCC and initiating ADT, were invited to participate. Eligibility criteria included age between 45 and 75 years, the ability to walk 400 m, medical clearance from their primary physician, ability to speak English, cognitive alertness (current Eastern Cooperative Oncology Group performance status available in the medical notes), sufficiently literate (education level available in the medical records), and ownership of a smartphone for >1 year. Men participating in rigorous structured exercise regimes (ie, seeing a professional trainer or training >5 days per week) in the last 6 months were excluded, as their inclusion could conflict with the app’s usability for documenting physical activity. Men with planned systemic chemotherapy, planned treatment with abiraterone or enzalutamide, bone metastases, acute illness, any musculoskeletal, cardiovascular, or neurologic disorders that would prevent them from exercising or put them at risk from exercising were excluded, as were men with hearing or visual impairment that would prevent them from participating in the focus group.

### Recruitment

Recognizing that the choice of an app-based intervention may skew the sample toward younger and well-educated patients, we provided access to the program for all patients at a cancer clinic as the point of service to reduce potential selection bias. This study took place over 2 months, beginning in March 2019. A convenience sample of 5 patients aged 45-75 years was recruited from the cancer clinic for a focus group discussion. Patients who were initiating ADT were recruited from 3 academic medical clinics affiliated with DF/BWHCC. Permission was sought from the patient’s primary care physician or oncologist before contacting them via phone. A research coordinator explained the rationale of the study, and the coordinator obtained verbal consent. The focus group interview was held at a mutually convenient time for all the participants in a hospital conference room. An additional sample of 5 physicians was recruited based on their specialties. Physicians who treat patients undergoing ADT were specifically contacted for individual in-depth interviews. The physicians were selected to gain insights on their beliefs about potential benefits of the mHealth app and whether they would routinely recommend it to their patients if it were readily available.

### Patient Focus Group

For the focus group, we used a pilot version of the app, which was available online on Google Play for Android phones and Apple Store for iPhones. Using the feedback from the usability testing, the developers later launched an updated version of the app. The coordinator followed a pre-established IRB–approved script to guide the focus group. Before initiating the discussion, the group introduced themselves, and then the study coordinator described the goals and expectations of the research project while asking clarifying questions (see Multimedia Appendix 1 for interview script). The focus group discussion was audio recorded and subsequently transcribed with the assistance of a transcription software, and the transcriptions were analyzed manually.

### Testing Usability and Definitions of Success

App usability was assessed through the participants’ responses while the focus group addressed specific scenarios. These scenarios included participants’ ability to register an online account and download the mHealth app, navigate options within the app, appreciate user-interface and engagement with the graphics of the mHealth app (worksheet available in Multimedia Appendix 1), and input information on their (1) typical physical activity in a given week, (2) recent exercise activity, and (3) recent meals (including the ability to take photos of their meals). All the participants used their own smartphones. Success was defined as the ability of the participants to complete the instructions in a given scenario. When instructions were executed completely, the response was considered “successful”; else the response was considered “unsuccessful.”

### Physician Interviews

Five physicians who treat patients with prostate cancer were identified and contacted. A multi-disciplinary physician cohort well versed in urology, medical oncology, and radiation oncology was recruited. In-depth interviews were scheduled and conducted in locations convenient for both the coordinator and the participant. The same IRB–approved script was used for each physician interview. The interviews were audio recorded and transcribed.

### Qualitative Analysis

The developed initial coding scheme was based on usability metrics from previous studies [12,13]. The transcripts were correlated with audio recordings of the patient focus group and physician interviews, and minor edits were made to the transcript (small talk or unrelated dialogue was removed). Once all the data were assembled, a master database of transcripts was compiled and read multiple times by JN and AV. A thematic content analysis was conducted. Significant and recurring themes (defined as themes appearing more than 3 times) were identified and coded in each transcript. Thematic discrepancies were resolved through consensus between the two coders. Each interview and focus group were summarized using these codes. These summaries produced recurring themes across all the data, which allowed us to iteratively refine the codebook and concept map. This cycle continued until all the significant themes were categorized and the transcripts and codebook were consistent with those themes.

## Results

### Baseline Characteristics

Table 1 shows the baseline characteristics of the patients and physicians recruited for this study.

**Table 1:** Participants’ characteristics (N=5).

Characteristics			Value
**Patients (N=5)**			
	Age (years), mean		62
	Gender – male, n (%)		5 (100)
	**Age (years), n (%)**		
		45-55	1 (20)
		56-65	2 (40)
		66-75	2 (40)
	**Medical conditions, n (%)**		
		Prostate cancer	5 (100)
	**Race/ethnicity, n (%)**		
		White	4 (80)
		Hispanic/Latino	1 (20)
	**Education status, n (%)**		
		Graduate/professional degree	2 (40)
		Some college	2 (40)
		GED^a^/high school diploma	1 (20)
**Physicians (N=5)**			
	**Specialty, n (%)**		
		Urologist	2 (40)
		Medical oncologist	2 (40)
		Radiation oncologist	1 (20)

^a^GED: General education development.

### Patient Focus Group Results

The focus group discussion with the participants covered the following dominant patient themes surrounding mHealth app usability: limited knowledge, participants’ appreciation for holistic care, and struggles with app registration and download. The focus group discussion also revealed that participants’ familiarity with mHealth apps was limited. This aspect was highlighted by statements such as, “There are so many options, wow.” Participants highly appreciated that health care organizations and physicians were proactively building digital solutions that would improve patient well-being and convenience, as evidenced by statements such as, “This sort of lets the patient know that there is more to us than just the cancer, there’s also our general well-being.” Last, participants were candid about the struggles they experienced while handling mHealth apps in general as patients with cancer, as revealed by statements such as, “It might just be me but it’s tough for me to understand.” Table 2 presents detailed statements on these themes.

**Table 2:** Participants’ reflections on the usage of the mHealth app.

Theme	Quotations
Participant knowledge	“There are so many options, wow – I didn’t realize that things that I do on a daily basis would be considered exercise. My wife would be so happy to hear that! What else did you ask? If it was intuitive? Yes, it is clear and intuitive to me.”
Participant appreciation	“I think it’s great that Dr.———, and whoever else is working on this study, cares about the—you know holistic care of the patient. This sort of lets the patient know that there is more to us than just the cancer, there’s also our general well-being.” “No, this is great. It shows that the providers care and that we are more than a number to them, I think.”
Participant struggling	“To be honest, I don’t think this is intuitive. It might just be me but it’s tough for me to understand.”

Participants’ responses to specific scenarios revealed that they were generally comfortable using the mHealth app for documenting exercise and diet. For Scenario 1, which tested the participants’ ability to register an online account and download the mHealth app, we found that 60% (3/5) were “successful” and 40% (2/5) were “unsuccessful.” For Scenario 2, which tested the participants’ ability to input information on their typical physical activity in a given week, we found that 100% (5/5) were “successful.” For Scenario 3, which tested the participants’ ability to navigate options within the app, we found that 100% (5/5) were “successful.” For Scenario 4, which tested the participants’ ability to input information on their recent exercise activity, we found that 60% (3/5) were “successful” and 40% (2/5) were “unsuccessful.” For Scenario 5, which tested the participants’ ability to input information on their recent meals as well as take photos of their meals, we found that 80% (4/5) were “successful” and 20% (1/5) were “unsuccessful.” Last, for Scenario 6, which tested the participants’ ability to appreciate the user-interface and engage with the graphics of the mHealth app, we found that 100% (5/5) were “successful.”

### Physician Interview Results

Among the health care physicians, the dominant themes reflected their concern regarding the undermining of patients, patients’ fear of technology, and the ability of older patients to access a smartphone app (Table 3). When queried about patients’ ability to employ technology for recording exercise and dietary activities, the physicians believed that most patients would not be able to do so, as expressed by their statements:

I mean, I bet some patients are tech savvy, but the majority of patients I see are not.

The interviews also revealed that the physicians were apprehensive that integrating technology as part of their health care would lead to increased stress among their patients:

I think patients will struggle with this. I think that we need to be careful with technological…interventions like this because it may make them more stressed.

We also found that the physicians believed that age would prevent older patients from benefitting from the advantages of using the mHealth app:

But I worry that the patients won’t actually use it. It might be hard for older patients, and most prostate cancer patients are above the age of 45.

In addition to these impressions, the physicians also emphasized the intuitiveness and ease of navigating the app, as indicated by statements such as, “…it’s easy to go back and forth between tabs” and “It’s a great app. The design is simple, intuitive, and professional.”

**Table 3:** Physicians’ reflections on the usage of the mHealth app.

Theme	Quotations
Undermining patients	“I think my con is that, I don’t think older patients, or any patients with prostate cancer will understand how to use this app.” “I mean, I bet some patients are tech savvy, but the majority of patients I see are not.”
Patients’ fear of technology	“I think patients will struggle with this. I think that we need to be careful with technological…interventions like this because it may make them more stressed.”
Concerns for older patients	“The app is really well designed and is…generally pretty intuitive. But I worry that the patients won’t actually use it. It might be hard for older patients, and most prostate cancer patients are above the age of 45.”

## Discussion

### Findings

Mobile health apps present a unique opportunity to enhance patient engagement and self-management of chronic diseases. In this study, we found that the overall usability of an mHealth app in patients with prostate cancer participants on ADT was acceptable. Moreover, we found that participants generally appreciated their care teams’ efforts in devising technological solutions that would enhance their ability to record and monitor their diet and exercise. We also observed that physicians were generally skeptical of the benefits of integrating technology with conventional health care to augment prostate cancer care. A disconnect was found between the experiences of the participants and the physicians on the potential of health technology solutions for improving prostate cancer care.

The findings of our study have several implications. First, we found that the participants were generally successful in using the mHealth app and were able to input data about their meals and exercise. Most participants were able to navigate through various features of the app (ie, move between the tabs and the home screen) and take photos of their meals. Our findings contrast those of Sarkar et al [14], who found that the usability and applicability of mHealth apps for self-management of chronic conditions was limited [14]. Sarkar et al [14] examined the acceptability and usability of mHealth apps in a set of racially diverse and economically disadvantaged patients—communities that often have higher prevalence of chronic diseases (diabetes and depression) and require caregiving. They also examined usability by asking participants to perform specific tasks on 11 apps. While our study also examined usability by asking participants to perform a variety of tasks, we had a more specific focus, namely, the participants’ ability to record exercise and diet, interventions that can impact treatment, and disease outcomes. In addition, Sarkar et al [14] attempted to understand usability among existing apps, while our goal was to document how a newly developed app can be improved for greater acceptance among patients. We found that the participants appreciated that they could use the mHealth app for tracking their typical physical activity—an important consideration for patients who may develop sarcopenia. This finding correlates with those of previous studies reporting that prostate cancer patients are often receptive to using mHealth apps to promote physical activity, especially if the technology is easy to use [15]. ADT is associated with decreased bone mineral density and higher risk of fracture, and current evidence indicates that improvements in dietary intake (calcium and vitamin D supplementation) could alleviate these risks [16]. Our analysis underscores the benefit of using mHealth apps to record and track dietary intake. Additionally, patients on ADT are at risk of developing several metabolic adverse events, including weight gain and diabetes [7,16]. Moreover, given that mHealth apps can record the level of physical activity, these online tools can potentially mitigate the impact of metabolic adverse events.

Second, we found that our sample of physicians was not convinced that using mHealth technology for prostate cancer patients would be beneficial, although they remarked positively on its general usability, appearance, and navigation. These perceptions were based on their beliefs that patients lacked technological understanding, would be unable to use mHealth apps, and their advanced age would preclude them from benefitting from technological interventions. This finding is in conflict with those of a previous work as well as the results of patients’ usability evaluations from this study. Nguyen et al [17] reported that physicians viewed patient-focused apps positively and believed that these interventions would augment their ability to provide patient care. The same study also observed that physicians believed that using mHealth apps would supplement their role by providing additional medical information to their patients electronically and therefore enhance self-management of chronic conditions.

Third, our findings point to the role of physician specialization as a determinant of mHealth app adoption. As per Bodur et al [18], medical and nursing students believed that health technology could play an increasingly important role in the delivery of care in the future [18]. It is possible that these differences are driven by variations in clinical focus; we interviewed prostate cancer specialists, and previous studies have mostly evaluated general practitioners. Although there is evidence that cancer specialists are highly satisfied with using mHealth technology for improving cancer care delivery, it seems that specialists perceive a greater benefit of adopting mHealth apps at the population, rather than the patient, level [19].

Last, our analyses also reveal the various factors that can influence the adoption of mHealth apps. Our qualitative analyses underscore how ease of use and registration/download, intuitive appearance of the app, ability to navigate various tabs within the app, and focus on holistic cancer care are important adoption considerations for patients as well as physicians. These findings concur with previous analyses. In a comprehensive review, Alam et al [20] reported that factors such as performance expectancy (the belief that technology will achieve expected outcomes), effort expectancy (ease of use), and facilitating conditions (the belief that technology/organization infrastructure supports usage) were positively associated with greater adoption of mHealth technology [20]. Our study provides additional evidence to support incorporation of these usability features in mHealth apps for patients.

Given the methodological approach we pursued, our study has several limitations. We studied a small sample of physicians and patients from a single cancer center. The results may vary with larger–scale implementation. We evaluated the participants in our study on a specific mHealth app and provided the service free of charge, which may lead to selection bias and limit the generalizability of this work, respectively. While some mHealth apps provide information on prostate cancer, a recent systematic review revealed that most commercially available apps do not provide culturally sensitive information and have major usability concerns [21]. Other studies have reported that patients would be willing to pay for these services—especially if they are affordable [22,23]. In our study, the patient participants were aware that their responses were being recorded and evaluated, which could have led to changes in observed behavior [24] and overestimated the usability of the app due to social desirability bias. Although the participants were aware that their own care teams were involved in this study, results may vary when implementing such mHealth apps in a different context. Moreover, we did not evaluate the efficacy of the app to improve prostate cancer outcomes; we chose to first evaluate the usability and feasibility of the app, as we viewed these aspects as necessary requirements for future studies on efficacy evaluation.

### Conclusion

Usability was observed to be an important factor for adoption of the proposed mHealth technology, as determined by ease of registration and use, intuitive appearance of the app, and focus on holistic cancer care. Men receiving ADT for prostate cancer treatment had an overall positive experience using the mHealth app to record and track their diet and exercise. The physicians believed that the app was easy to use but raised concerns about usability among older men who do not commonly use smartphone apps. This work provides foundational evidence for studies that examine the feasibility, efficacy, and interoperability of using mHealth apps for prostate cancer patients.

## Appendix

Multimedia Appendix 1 Usability test.

## Abbreviations

ADT: Androgen Deprivation Therapy
DF/BWHCC: Dana-Farber/Brigham and Women’s Cancer Center
GED: General Education Development
IRB: Institutional Review Board
mHealth: mobile health

## References

[1] Greene J, Hibbard JH, Sacks R, Overton V, Parrotta CD (2015). When patient activation levels change, health outcomes and costs change, too. Health Aff (Millwood).

[2] Broderick A, Haque Farshid (2015). Mobile health and patient engagement in the safety net: a survey of community health centers and clinics. Issue Brief (Commonw Fund).

[3] Cortez NG, Cohen IG, Kesselheim AS (2014). FDA regulation of mobile health technologies. N Engl J Med.

[4] Berry DL, Hong F, Halpenny B, Partridge AH, Fann JR, Wolpin S, Lober WB, Bush NE, Parvathaneni U, Back AL, Amtmann D, Ford R (2014). Electronic self-report assessment for cancer and self-care support: results of a multicenter randomized trial. J Clin Oncol.

[5] Plante TB, Urrea B, MacFarlane ZT, Blumenthal RS, Miller ER, Appel LJ, Martin SS (2016). Validation of the instant blood pressure smartphone app. JAMA Intern Med.

[6] Steinhubl SR, Muse ED, Topol EJ (2013). Can mobile health technologies transform health care?. JAMA.

[7] Smith Matthew R, Finkelstein Joel S, McGovern Francis J, Zietman Anthony L, Fallon Mary Anne, Schoenfeld David A, Kantoff Philip W (2002). Changes in body composition during androgen deprivation therapy for prostate cancer. J Clin Endocrinol Metab.

[8] Dawson JK, Dorff TB, Todd Schroeder E, Lane CJ, Gross ME, Dieli-Conwright CM (2018). Impact of resistance training on body composition and metabolic syndrome variables during androgen deprivation therapy for prostate cancer: a pilot randomized controlled trial. BMC Cancer.

[9] Grbich C (2010). Qualitative data analysis: An introduction. Researching Practice.

[10] Morgan DL (1993). Qualitative content analysis: a guide to paths not taken. Qual Health Res.

[11] Bloor M, Wood F (2006). Keywords in qualitative methods: A vocabulary of research concepts.

[12] Taha J, Sharit J, Czaja SJ (2014). The impact of numeracy ability and technology skills on older adults' performance of health management tasks using a patient portal. J Appl Gerontol.

[13] Segall N, Saville Jeffrey G, L'Engle Pete, Carlson Boyd, Wright Melanie C, Schulman Kevin, Tcheng James E (2011). Usability evaluation of a personal health record. AMIA Annu Symp Proc.

[14] Sarkar U, Gourley Gato I, Lyles Courtney R, Tieu Lina, Clarity Cassidy, Newmark Lisa, Singh Karandeep, Bates David W (2016). Usability of commercially available mobile applications for diverse patients. J Gen Intern Med.

[15] Roberts AL, Potts HW, Koutoukidis DA, Smith L, Fisher A (2019). Breast, prostate, and colorectal cancer survivors' experiences of using publicly available physical activity mobile apps: Qualitative study. JMIR Mhealth Uhealth.

[16] Nguyen Paul L, Alibhai Shabbir M H, Basaria Shehzad, D'Amico Anthony V, Kantoff Philip W, Keating Nancy L, Penson David F, Rosario Derek J, Tombal Bertrand, Smith Matthew R (2015). Adverse effects of androgen deprivation therapy and strategies to mitigate them. Eur Urol.

[17] Nguyen AD, Frensham LJ, Baysari MT, Carland JE, Day RO (2019). Patients' use of mobile health applications: what general practitioners think. Fam Pract.

[18] Bodur G, Gumus S, Gursoy NG (2019). Perceptions of Turkish health professional students toward the effects of the internet of things (IOT) technology in the future. Nurse Educ Today.

[19] Sharma JJ, Gross G, Sharma P (2012). Extending oncology clinical services to rural areas of Texas via teleoncology. J Oncol Pract.

[20] Alam MZ, Hoque MR, Hu W, Barua Z (2020). Factors influencing the adoption of mHealth services in a developing country: A patient-centric study. International Journal of Information Management.

[21] Owens OL, Beer JM, Reyes LI, Thomas TL (2019). Systematic review of commercially available mobile phone applications for prostate cancer education. Am J Mens Health.

[22] Ebner C, Wurm Elisabeth Mt, Binder Barbara, Kittler Harald, Lozzi Gian Piero, Massone Cesare, Gabler Gerald, Hofmann-Wellenhof Rainer, Soyer H Peter (2008). Mobile teledermatology: a feasibility study of 58 subjects using mobile phones. J Telemed Telecare.

[23] Pathipati AS, Ko JM (2016). Implementation and evaluation of Stanford Health Care direct-care teledermatology program. SAGE Open Med.

[24] Borycki E, Monkman Helen, Griffith Janessa, Kushniruk Andre W (2015). Mobile usability testing in healthcare: Methodological approaches. Stud Health Technol Inform.

