# Supplementary material for: Mobile Health App for Prostate Cancer Patients on Androgen Deprivation Therapy: Qualitative Usability Study
Source: JMIR Mhealth Uhealth. 2020 Nov 3;8(11):e20224. doi: 10.2196/20224 (PMC7671847; doi:10.2196/20224)
Supplement: Multimedia Appendix 1 [file mhealth_v8i11e20224_app1.pdf]

Usability Test:

**App download and Registration:**<br>You have received an email from the Research Coordinator regarding the utilization of a mobile health app to help men with prostate cancer about to initiate androgen deprivation therapy (ADT) engage in physical activities and better eating habits.<br><br>We are interested in getting your feedback on how easy or how hard the mobile health app is to use, and whether the things you will read are easy or hard to understand.<br><br>This is not a test. There are no right or wrong answers. We simply want to make sure that the program is usable and makes sense to you. If you struggle to fin something, or if something doesn't make sense, let me know.<br><br>Talking aloud as you read through the questions and go from screen to screen on the app will help us learn from you and improve the program. Remember: if you run into difficulties, it's because we need to improve the app, so please tell us how we can make it better. It is not possible for you to say something wrong.<br><br>All comments will be kept confidential as to who provided them. By not sharing this information with those beyond the investigators of this study, we hope this will encourage you to speak freely.<br>This session will take about 30 minutes, and we will take notes.<br><br>Let's get this started.	**User feedback:**
**Scenario 1:**<br>Imagine that you are being invited to use this app after learning you have prostate cancer and about to start treatment with ADT. The app is to help you engage in	**User feedback:**

more physical activities in your weekly routine and to help you develop better eating habits.<br><br>First you will receive the following email requesting you to register via our online server. Please read through the email and talk us through what you would do next after receiving this email, including whether you find the instructions well-described, and easy to follow.<br><br>Then please go on to search for the app and downloading it based on the instructions provided within that email, and let me know if you run into any trouble with that part.<br><br>As the app is downloaded, please register as you would based on the instructions provided within that email, and let me know if you run into any trouble or if there is anything that is hard to understand.	
**Scenario 2:**<br>Next, the app will ask you what typical physical activities you will engage in during the week. What do you do next?<br>Is it clear from the screen that you may change these options any time?<br>Are you able to find the types of physical activities you engage in from the provided list?<br><br>Feel free to continue talking out these questions and answer choices, and to give us your thoughts.	**User feedback:**
**Scenario 3:**<br>As you are taken to the home screen of the app, please familiarize yourself with the various facets of the app, such as the exercise activities tab, and the food log tab. How easy is it to navigate from one facet to another? Is navigation within the app intuitive?	**User feedback:**
**Scenario 4:**	**User feedback:**

Now please go to the exercise activities tab and pretend to log two of any activities from the options you have chosen.<br>How easy do you find logging exercise to be?<br>Did you realize that you can log exercise for the previous day?	
**Scenario 5:**<br>Now pretend that you want to record the food you ate.<br>Is it obvious that you can take a picture of the food, but that you can also select a previously taken picture from your camera roll?<br>What do you think of the food-rating process? Is the process intuitive?<br>Did you realize that you can record food you ate previously?	**User feedback:**
**Scenario 6:**<br>Please familiarize yourself with the graphics of the exercise and food tabs. Is it easy or difficult to understand the graphics?<br>Do you think anything can be improved?	**User feedback:**
**Wrap-up:**<br>Thank you for working your way through the app, and helping us identify areas where we can improve it.<br><br>In closing, would you please tell us what you liked best about the mobile health app? And least?<br><br>What do you think was the main purpose of the app?<br><br>Do you think the app will be helpful for men with prostate cancer?<br><br>Do you have any other feedback or suggestions that would help us improve the questionnaire or your experience with it?<br><br>Thank you for your time.	**User feedback:**
